# Circulating vascular biomarkers in relation to physiological indices of aortic stiffness and endothelial function in hypertension

**DOI:** 10.1038/s41598-025-25475-6

**Published:** 2025-10-28

**Authors:** Andreas Jekell, Mikael Ekholm, Thomas Kahan, Kristina Lundwall

**Affiliations:** 1 Division of Cardiovascular Medicine, Department of Clinical Sciences, Karolinska Institutet, Danderyd Hospital, Stockholm, Sweden; 2 Wetterhälsan Primary Care Centre, Region Jönköping County, Jönköping, Sweden

**Keywords:** Endothelial glycocalyx, Vascular biomarkers, Endothelial function, Aortic stiffness, Hypertension, Chronic kidney disease, Physiology, Prognostic markers, Renovascular hypertension, Chronic kidney disease

## Abstract

Studies investigating the relation between circulating vascular biomarkers reflecting endothelial dysfunction and physiological methods to evaluate vascular function remain limited. We simultaneously evaluated the relation between circulating endothelial biomarkers with physiological non-invasive vascular methods in 107 hypertensive patients with a wide range of mean estimated glomerular filtration rate (eGFR). Endothelial glycocalyx hyaluronan (HA) and syndecan-1 (SDC-1), and cellular adhesion molecules (ICAM-1, VCAM-1, and E-selectin) were measured by enzyme-linked immunosorbent assays. Aortic stiffness (cfPWV) was assessed by pulse wave analysis. Endothelial function in different vascular beds was evaluated physiological by methods: flow mediated vasodilation (large arteries), pulse wave analysis and the reflection index change, using beta 2-adrenoceptor agonist stimulation (smaller resistance arteries), and laser Doppler fluxmetry and iontophoresis (skin microvascular function). Diastolic function and left atrial size were assessed by echocardiography. Mean blood pressure (BP) was 149 ± 17/87 ± 10 mm Hg, mean eGFR 74 (21–130) ml/min x 1.73 m^2^. HA was independently related to cfPWV (β = 0.23, *P* = 0.02), whereas SDC-1 was independently inversely related to skin microvascular function, (β= − 0.27, *P* = 0.042) and was independently related to resistance artery endothelial function (β = 0.29, *P* = 0.026). All circulating biomarkers were unrelated to physiologically measured large artery endothelial function, and with echocardiographic parameters. The glycocalyx markers were associated with physiological vascular measures independent of eGFR but should not be considered as a proxy for these measures, due to the weak relations. However, combining circulating markers with physiological measures might give additional information about vascular function.

**Supplementary Information:**

The online version contains supplementary material available at 10.1038/s41598-025-25475-6.

## Introduction

Increased aortic stiffness as assessed by non-invasive carotid-femoral pulse wave velocity (cfPWV) or by augmentation index (AIx) is associated with worse prognosis for cardiovascular (CV) disease, and indices of aortic stiffness improves risk prediction for CV events and all-cause mortality in patients at intermediate risk^1,2^. Aortic stiffness seems to be an important mediator for the development of chronic kidney disease (CKD) in hypertension^3,4^, where changes in pulse wave propagation and wave reflection predict progression of renal and cardio-renal disease^5,6^.

Vascular endothelial dysfunction is a well-recognized risk marker for atherosclerotic disease, but the role in development of kidney injury in hypertension is less understood. Evaluation of endothelial function in the peripheral circulation can be performed by established methods^7,8^. Endothelial dysfunction of conduit arteries, measured non-invasively as flow-mediated vasodilation (FMD) of the brachial artery, is considered an independent predictor for atherosclerotic disease progression and impaired FMD also predict CV risk^9,10^. Forearm blood flow measured invasively by venous occlusion plethysmography measures mainly forearm skeletal muscle resistance artery function and an impaired endothelial function is a marker of CV risk in the general population and in hypertension^11,12^. Evaluation of endothelium dependent vasodilation of smaller resistance arteries can be assessed non-invasively by using pulse wave analysis (PWA) with applanation tonometry to measure changes of the reflective pulse before and after beta 2-adrenoceptor agonist stimulation^13,14^ Impaired skin microvascular endothelial function is believed to be a marker of importance in hypertension and in CKD^15,16^, and can be measured by Laser Doppler fluxmetry (LDF) and post occlusive reactive hyperemia, or by LDF and local administration of acetylcholine (ACh) and sodium nitroprusside (SNP) by iontophoresis^17^. Skin microvascular endothelial dysfunction may precede the development of endothelial dysfunction in larger arteries and may therefore serve as a biomarker of future CV risk^18^.

Several circulating vascular biomarkers, reflecting different aspects of endothelial dysfunction have emerged. Elevated concentrations of soluble adhesion molecules are present in different conditions, including heart failure with preserved ejection fraction and CKD^19,20^. The endothelial glycocalyx (eGCX), the outermost layer of the luminal surface adjacent to the vascular endothelium, is constituted by proteoglycans, glycoproteins, glycolipids, and glycosaminoglycans, with important regulatory functions to maintain the endothelial integrity and vascular homeostasis^21^. Degradation of the eGCX is associated with vascular inflammation, enhanced leukocyte adhesion and accelerated atherosclerosis in vitro, and coincides with an early phase of endothelial dysfunction^22,23^. The function and structure of the eGCX can be assessed either by visualization of the eGCX layer thickness^24^, or indirectly by measuring circulating degrading fractions in plasma, i e, shedding^25^. Increased shedding of syndecan-1 (SDC-1), a transmembrane proteoglycan, has been related to ischemia-reperfusion injury in clinical conditions associated with increased oxidative- and vascular shear stress causing eGCX degradation^26^. Thus, increased shedding of the eGCX structural surface core proteins might be taken to serve as surrogate biomarkers of endothelial integrity.

Studies on the relation between circulating vascular biomarkers and physiological methods for evaluating arterial stiffness and endothelium dependent vascular function remain limited. Such knowledge is important to help understanding the role of circulating biomarkers in the assessment of vascular function and, potentially, for clinical risk stratification^27^. This is especially important in vascular risk populations such as patients with hypertension and kidney dysfunction. In this study we therefore investigate simultaneously the relation between circulatory biomarkers of vascular function and physiological methods to evaluate vascular function in patients with hypertension and a wide range of kidney function, a novel approach that to our knowledge has not yet been performed. Given the limited availability of data on the concurrent evaluation of endothelial biomarkers and vascular physiology in this context, this study aims to address a critical gap in understanding the relationship between circulating vascular biomarkers and physiological measures of vascular function.

## Results

### General

Clinical characteristics of the study population are presented in Table 1. Middle aged women and men with mild-to-moderate hypertension were included. About one third were on antihypertensive treatment. The subjects were slightly overweight, normoglycemic, and did not exhibit severe hyperlipidaemia. Few were smokers. Mean estimated glomerular filtration rate (eGFR) was 74 (range 130 − 21) ml/min x1.73 m^2^, and 36% had an eGFR > 90 ml/min x1.73 m^2^. Indices of arterial stiffness, vascular endothelial function in various vascular beds, and echocardiographic measurements are presented in Table 2.

**Table 1:** Baseline characteristics.

Age, years (range)	58 ± 13 (23–87)
Male sex, n	73
Height, cm	175.5 ± 8.8
Body mass index, kg/m^2^	26.8 ± 4.3
Current smoking, n	6
Systolic BP, mm Hg	149.1 ± 16.6
Diastolic BP, mm Hg	87.3 ± 10.1
Heart rate, bpm	58.3 ± 7.4
Ongoing BP treatment	33 (31)
ACEi or ARB	29 (27)
Beta blockers	18 (17)
Calcium channel blockers	22 (21)
Other	12 (11)
eGFR, ml/min *x* 1.73m^2^	73.4 ± 27.4 (21–130)
uACR, mg/mmol	13.7 ± 38.8
Glucose, mmol/l	5.5 ± 0.6
Total cholesterol, mmol/l	5.1 ± 1.1
LDL cholesterol, mmol/l	3.3 ± 0.9
Hyaluronan (ng/ml)	15 [11–20]
Syndecan-1 (ng/ml)	38 [27–54]
ICAM-1 (ng/ml)	302 [264–356]
VCAM-1 (ng/ml)	347 [304–408]
E-selectin (ng/ml)	32 [26–41]

Data presented as mean values ± SD or as median and interquartiles, for 107 patients. Ongoing antihypertensive treatment for SOLID study subjects, presented as n (%). In DoRa, all study subjects were untreated, with no antihypertensive medication or stiatin treatment.

*BP* blood pressure, *ACEi* angiotensin converting enzyme inhibitor, *ARB* angiotensin receptor blocker, *eGFR* estimated glomerular filtration rate, *uACR* urine albumin-creatinine ratio,* LDL* low density lipoprotein, *ICAM-1* intracellular adhesion molecule-1, *VCAM-1* vascular cell adhesion molecule-1.

**Table 2:** Indices of aortic stiffness, endothelial function, skin microvascular function, and LV diastolic function and left atrial size.

cPP (mm Hg)	52 ± 14.8
AIx (%)	30.3 ± 11.5
cfPWV (m/s)	9.4 ± 2.8
crPWV (m/s)	9.0 ± 1.2
cfPWV / crPWV	1.0 ± 0.3
FMD (%)	5.4 ± 4.2
GTN (%)	16.0 ± 7.0
RI (Δ%)	–7.0 ± 3.0
ACh peak flux (PU)	33 [19–61]
SNP peak flux (PU)	55 [37–82]
E/e’	8.9 ± 2.4
LAVI (ml/m^2^)	16.3 ± 5.0

Data is presented as mean values ± SD, or as median and interquartiles for 107 patients.

*cPP* central pulse pressure, *AIx* augmentation index, *cfPWV* carotid to femoral pulse wave velocity, *crPWV* carotid to radial pulse wave velocity, *FMD* post-ischemic forearm flow-mediated vasodilation, *GTN* glytrine trinitrate, i.e. endothelium independent vasodilation; RI (Δ%), reflection index change (i.e. the relative change in height of the diastolic reflecting pulse wave before and after beta 2-adrenoceptor agonist stimulation), *ACh peak flux* acetylcholine induced forearm skin flow reactivity; SNP peak flux, sodium nitroprusside induced forearm skin flow reactivity, *PU* perfusion units, *E/e’* peak velocity flow in early diastole (E) divided by mitral annular early diastolic velocity (e’), *LAVI* left atrial volume index.

### Circulating vascular biomarkers in relation to physiological indices of aortic stiffness

**Fig. 1:**
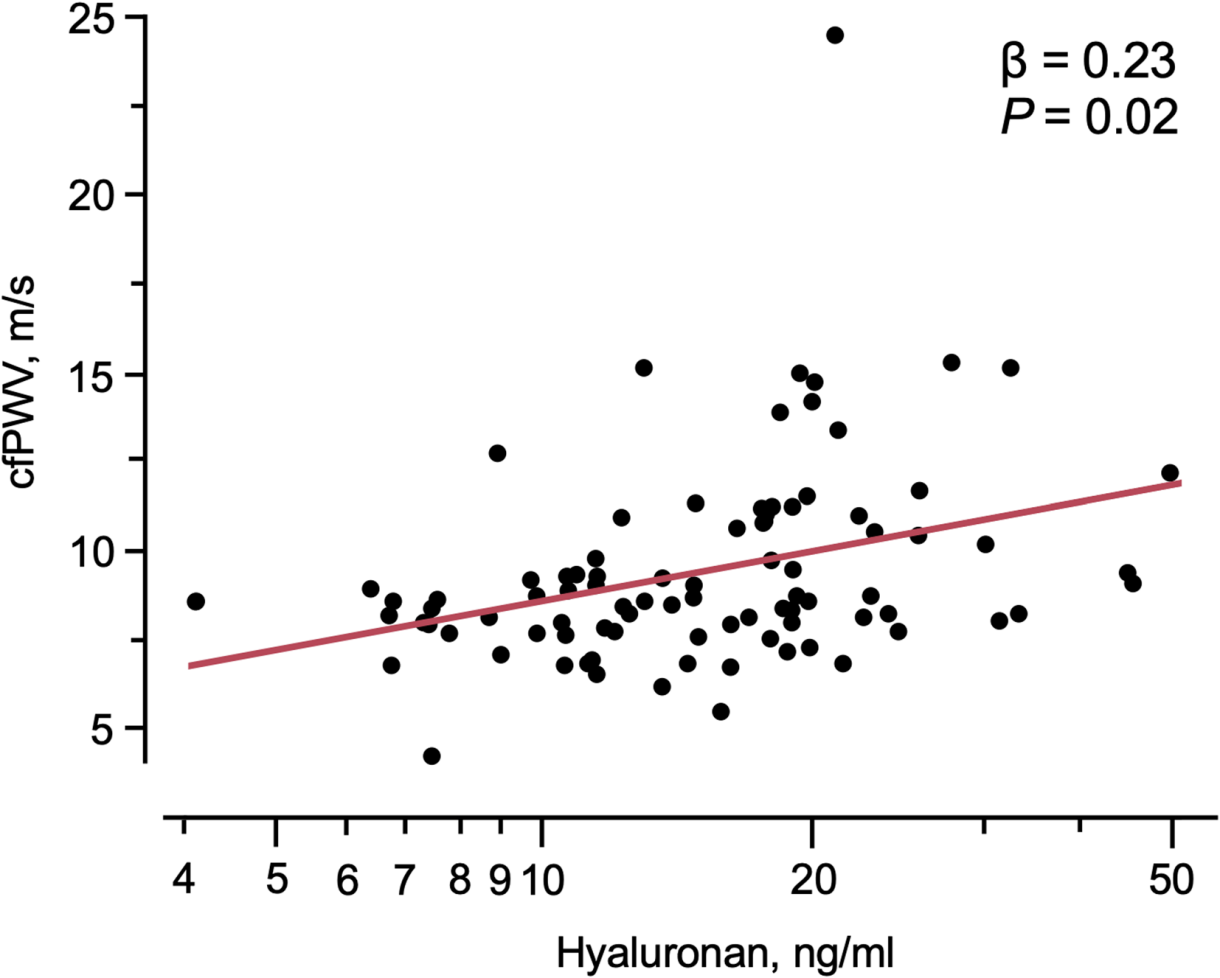
The relation between carotid-femoral pulse wave velocity (cfPWV) and hyaluronan. The beta coefficients (β) and significance levels (P) for the multivariable linear regression model including age, sex, MAP, ongoing BP treatment and eGFR in 89 patients are given. Hyaluronan was log transformed.

Results are presented in Table 3 and in Fig. 1. VCAM-1 related to cfPWV, and E-selectin to central pulse pressure (cPP) by bivariate correlation (Table 3). However, these relations were not retained in multivariable analyses including age, sex, mean arterial pressure (MAP), ongoing BP treatment, and eGFR. The eGCX marker hyaluronan (HA) related to cfPWV, Aix, and cPP by bivariate analyses (Table 3). In multivariable analyses, only the relation between HA and cfPWV was preserved (Table 3; Fig. 1).

**Table 3:** Relations between Circulating vascular biomarkers and physiological indices of aortic stiffness, LV diastolic function and left atrial size, and estimated kidney function bivariate and multivariable regression analyses adjusted for age, sex, MAP, ongoing BP treatment and eGFR in 74–102 patients.

		E-selectin	ICAM-1	VCAM-1	Hyaluronan	Syndecan-1
cfPWV	R, Pearson	-0.12	0.03	0.25	0.34	-0.05
	p, bivariate	0.27	0.82	0.03	0.001	0.68
	beta	n/a	n/a	0.18	0.23	n/a
	p multivariable	n/a	n/a	0.15	0.02	n/a
	n	82	74	74	89	85
AIx	r (Pearson)	-0.12	0.08	0.02	0.25	-0.13
	p	0.3	0.5	0.8	0.011	0.21
	beta	n/a	n/a	n/a	-0.11	n/a
	p multivariable	n/a	n/a	n/a	0.26	n/a
	n	95	89	89	102	98
cPP	r (Pearson)	-0.25	0.01	0.09	0.28	-0.14
	p	002	0.89	0.40	0.004	0.17
	beta	-0.11	n/a	n/a	0.04	n/a
	p multivariable	0.19	n/a	n/a	0.67	n/a
	n	94	88	88	101	97
LAVI	r (Pearson)	-0.18	-0.19	-0.08	0.27	-0.20
	p	0.09	0.09	0.46	0.008	0.06
	beta	n/a	n/a	n/a	0.22	n/a
	p multivariable	n/a	n/a	n/a	0.12	n/a
	n	90	83	83	96	92
E/e’	r (Pearson)	0.12	0.11	0.18	0.41	0.01
	p	0.27	0.32	0.09	< 0.001	0.93
	beta	n/a	n/a	n/a	0.11	n/a
	p multivariable	n/a	n/a	n/a	0.38	n/a
	n	93	87	87	100	97
eGFR	r (Pearson)	-0.01	0.06	-0.50	-0.45	-0.07
	p	0.91	0.57	< 0.001	< 0.001	0.50
	beta	n/a	n/a	-0.39	-0.04	n/a
	p multivariable	n/a	n/a	< 0.001	0.58	n/a
	n	98	90	90	105	101
uACR	r (Pearson)	0.19	-0.01	0.32	0.201	0.10
	p	0.07	0.90	0.003	0.05	0.36
	beta	n/a	n/a	0.14	0.13	n/a
	p multivariable	n/a	n/a	0.20	0.32	n/a
	n	89	82	82	95	92

*n/a* not applicable, *AIx* augmentation index, *cfPWV* carotid to femoral pulse wave velocity, *cPP* central pulse pressure, *ICAM-1* intracellular adhesion molecule-1, *VCAM-1* vascular cell adhesion molecule-1, *E/e’* peak velocity flow in early diastole (E) divided by mitral annular early diastolic velocity (e’), *LAVI* left atrial volume index, *eGFR* estimated glomerular filtration rate, *uACR* urine albumin-creatinine ratio.

To further evaluate predictors of elevated aortic stiffness, multivariable logistic regression models were constructed using cfPWV recommended cut-off ≥ 10 m/s and median-based stratification of ≥ 8.6 m/s with the same independent variables and covariates as the linear model. eGCX markers were not key determinants of high cfPWV. Results are presented in detail in Supplementary Tables 1 and 2.

### Circulating vascular biomarkers in relation to LV diastolic function and left atrial size by echocardiography

Results are presented in Table 3. HA related to both E/é and LA size in bivariate models, but these relations were not preserved in multivariable models. There were no other significant relations between circulating biomarkers, E/é and LA size.

### Circulating vascular biomarkers in relation to physiological assessment of large artery endothelial function

None of the investigated soluble adhesion molecules related to forearm endothelial dependent vasodilation assessed by FMD by bivariate analyses. Only ICAM-1 related weakly in bivariate analysis to endothelium independent vasodilation assessed by glyceryl trinitrate (GTN) (*r*= − 0.22, *p* = 0.04), but this association was not maintained significant by the multivariable analysis (*r*= − 0.20; *p* = 0.07). HA and SDC-1 were unrelated to both FMD and GTN.

### Circulating vascular biomarkers in relation to physiological assessment of endothelial function in smaller resistance arteries and of skin microvascular function

**Fig. 2:**
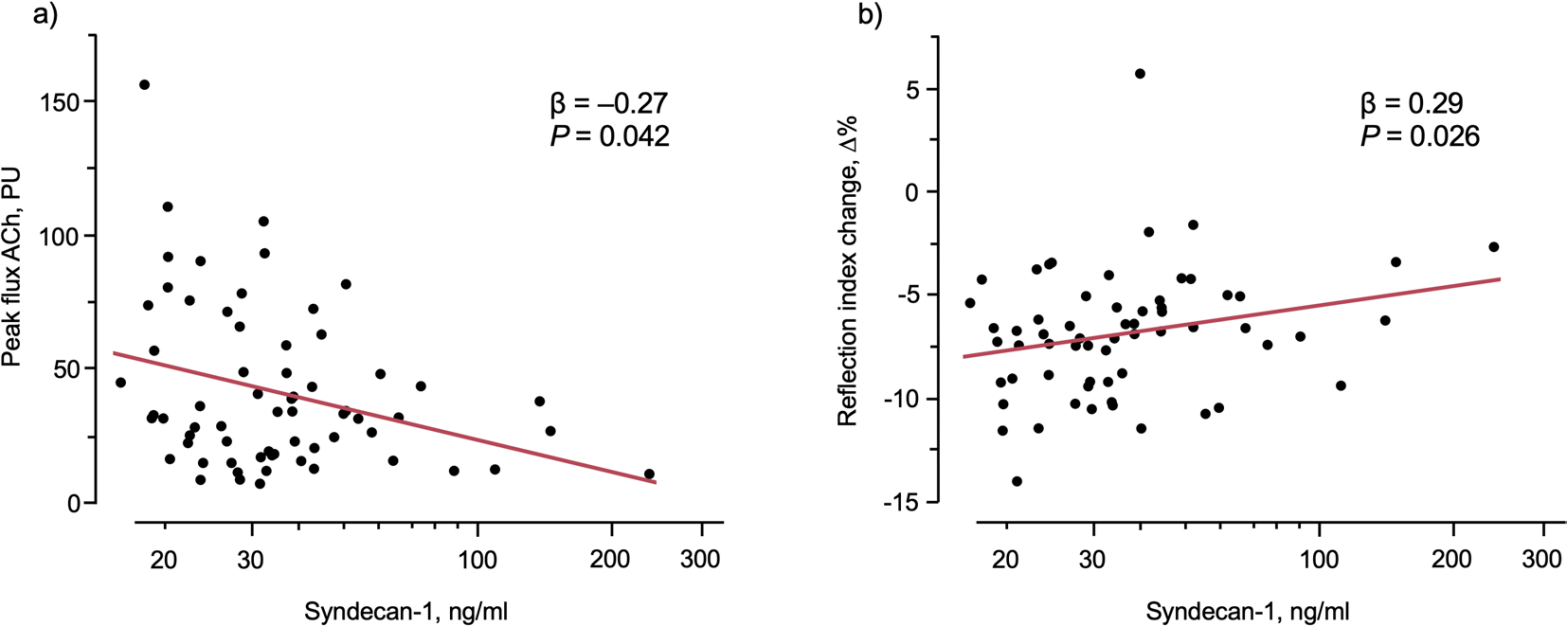
The relation between peak flux ACh (**a**), and the relative change in reflection index (Δ%) (**b**), and syndecan-1. The beta coefficients (β) and significance levels (P) for the multivariable linear regression model including age, sex, MAP, ongoing BP treatment and eGFR in 63–65 patients are given. Syndecan-1 was log transformed.

Results are presented in Table 4; Fig. 2a-b. There were no relations between soluble adhesion molecules and physiological assessment of endothelial function of smaller resistance arteries, evaluated by reflection index change (RIΔ%), or to skin microvascular function, evaluated by ACh peak flux, respectively. In contrast, eGCX marker SDC-1 related to endothelial function of smaller resistance arteries evaluated by RI Δ% and was inversely related to skin microvascular function evaluated by ACh peak flux in both bivariate and multivariable models, controlled for age, sex, MAP, ongoing BP treatment and eGFR (Table 4; Fig. 2a,b). There were no relations between HA and smaller artery endothelial function or with skin microvascular function. Neither adhesion molecules nor eGCX markers were related to SNP peak flux (data not shown).

**Table 4:** Relations between Circulating biomarkers and endothelial function in smaller resistance arteries and skin microvascular endothelial function.

		E-selectin	ICAM-1	VCAM-1	Hyaluronan	Syndecan-1
RI (Δ%)	r (Pearson)	0.19	0.17	-0.02	-0.06	0.25
	p	0.15	0.19	0.91	0.65	0.05
	beta	n/a	n/a	n/a	n/a	0.29
	p multivariable	n/a	n/a	n/a	n/a	0.026
	N	59	59	59	66	63
ACh peak flux	r (Pearson)	-0.2	-0.1	-0.1	0.2	-0.31
	p	0.1	0.5	0.5	0.1	0.012
	beta	n/a	n/a	n/a	n/a	-0.27
	p multivariable	n/a	n/a	n/a	n/a	0.042
	N	61	60	60	68	65

Bivariate and multivariable regression analyses adjusted for age, sex, MAP, ongoing BP treatment and eGFR in 59–68 patients.

*ACh* acetylcholine, *ICAM-1* intracellular adhesion molecule-1, *RI* reflection index, *VCAM-1* vascular cell adhesion molecule-1. *n/a* not applicable. Neither adhesion molecules nor eGCX markers were related to SNP peak flux (data not shown).

### Circulating vascular biomarkers in relation to measures of estimated kidney function

To further investigate the impact of kidney function on assessed biomarkers, regression analyses of relations to eGFR and urine albumin-creatinine ratio (uACR) were performed. Multivariable analyses were controlled for sex, age, ongoing BP treatment and MAP (eGFR excluded as it was used as dependent variable). Results are presented in Table 3.

Soluble adhesion molecule VCAM-1 showed bivariate relations to eGFR and uACR, but the multivariable analysis showed that only the relation to eGFR remained significant (β= -0.39, *p* < 0.001). The eGCX marker HA was also related to eGFR and uACR, but these relations were not maintained in multivariable analyses.

## Discussion

### Main findings

This appears to be the first study to report on the relation between simultaneously measured circulating vascular biomarkers and physiological assessment of vascular function in several vascular beds in patients with mild-to-moderate hypertension and various degree of decline in kidney function. The study design with simultaneous biochemical and physiological measurements provides a possibility to compare two approaches to assess vascular function and reactivity without potential fluctuations in confounding factors. Our findings indicate generally weak associations. However, the eGCX marker HA was related to indices of aortic stiffness, and the eGCX marker SDC-1 to physiological assessment of endothelial dysfunction of smaller resistance arteries and skin microvascular dysfunction, independent of age, sex, mean BP, ongoing antihypertensive medication, and eGFR.

This study shows a relation between soluble circulating HA and aortic stiffness (assessed by cfPWV), an established marker for worse cardiovascular prognosis, independent of known confounding factors such as age and eGFR^2^. Increased vascular stiffening may be explained by low shear stress of the arterial wall, which activates hyaluronidase, resulting in shedding of HA of the eGCX-layer and loss of its regulatory and protective role of the vascular endothelium causing vascular inflammation and accelerated atherosclerosis^28^. There are, however, conflicting findings regarding the relation between measures of arterial and aortic stiffness and eGCX markers. One study showed that reduced eGCX layer was present in patients with cancer, but cfPWV was similar comparing cancer patients with controls^29^. In a cross-sectional multi-ethnic community-based study, including patients with traditional risk factors or history of CV disease there were associations between reduced eGCX size (increased perfused boundary region) with female sex and diabetes, but no associations between eGCX size and prevalent CV disease^30^. However, our findings suggesting that circulating HA levels independently relate to aortic stiffness align with studies in hypertensive patients where a thinner eGCX-layer measured by the perfused boundary region in the sublingual microcirculation associate with increased arterial stiffness and coronary microvascular dysfunction^31^. We extend these findings to suggest that also shedding of HA to the blood stream is related to measured cfPWV in subjects with hypertension, independent of age and level of eGFR.

Furthermore, using the same independent variables and covariates as for linear regression, we evaluated predictors for higher cfPWV using recommended clinical cut-off value (≥ 10 m/s)^32^. For a comparison a median-based stratification (≥ 8.6 m/s) was performed. These findings show that age, blood pressure, and sex are key determinants of increased aortic stiffness, whereas eGCX markers are not.

We found no association between soluble adhesion molecules and large artery endothelial function. Other studies report inconclusive results in healthy control subjects^33,34^, whereas expression of adhesion molecules in patients with diabetes and with Marfan syndrome is related to reduced FMD^35,36^. Our results are in contrast with a study in patients with antiphospholipid syndrome showing impaired FMD, increased shedding of SDC-1, and a thinner eGCX-layer^37^. This may in part be due to the different study populations. Also, one third of our patients were on antihypertensive treatment, which may have influenced both expression of vascular biomarkers and large artery endothelial function. However, this may appear less likely as our results were controlled for ongoing antihypertensive treatment (albeit present in only one third of our patients), without significant impact on the results.

One factor that contribute to this lack of correlation may be the inherent difference between global and regional assessments of vascular function. Circulating biomarkers may reflect systemic endothelial activation or injury, not directly associated with functional changes in distinct vascular segments. Furthermore, the interpretation of these biomarkers is dependent on both production and clearance mechanisms, which introduces variability. In contrast, non-invasive physiological methods provide direct, but segment-specific insights into endothelial performance.

In line with this, the interrelationship between physiological methods assessing endothelial function across different vascular beds is limited, as demonstrated by our group and others^38–41^. Also, we have previously reported results from the current cohort showing that endothelial function in large arteries, as evaluated by FMD, show no associations with endothelial function in smaller resistance arteries or the skin microcirculation^38^. Taken together, these findings show the complexity of vascular assessment and highlight the need for multimodal approaches to capture the heterogeneity of endothelial dysfunction across different vascular beds.

Circulating SDC-1 levels in this study were related to physiological measures of impaired endothelial function in smaller resistance arteries, independent of age, sex, MAP, ongoing BP treatment, and eGFR. In smaller resistance arteries the endothelium dependent vasodilation is mediated through local release of nitric oxide where SDC-1 is an important regulatory core glycoprotein for endothelial NO synthesis. The increased shedding of SDC-1 might be induced by changes in intravascular shear stress with degradation of the endothelial surface layer.

SDC-1 was independently inversely related to ACh-mediated peak flux as a physiological measure of skin microvascular function. ACh-mediated peak flux represents the endothelium-dependent vasodilation in skin microcirculation, mainly mediated through a delayed nitric oxide release^17^. In contrast to ACh-mediated peak flux, the SNP-mediated, and heat induced peak flux, representing the endothelial independent vasodilatation, was unrelated to SDC-1 (data not shown).

Degradation of the eGCX has been associated with microvascular dysfunction in various vascular beds. This includes the early onset of preeclampsia^42^, its association with indices of coronary microvascular dysfunction in patients with suspected coronary artery disease^43^, its correlation with poorer prognosis in acute myocardial infarction^44^, and its connection to microvascular dysfunction in patients with septic shock^45^. Other studies indicate an impaired eGCX in CKD, with associations between increased shedding of SDC-1 and HA and thinner eGCX-layer in the sublingual microvasculature in patients with polycystic kidney disease^46^. Our study adds novel results in hypertensive patients, showing soluble SDC-1 to be related to physiological indices of impaired endothelial function of smaller resistance arteries and to skin microvascular dysfunction, independent of factors of known importance for vascular function, such as kidney function and age.

In patients with CKD an increased shedding of SDC-1 and HA related with worsening stage of CKD^47^. However, in an elderly community cohort the association between directly measured endothelium-dependent and endothelium independent vasodilatation in resistance arteries evaluated with the invasive forearm technique (using ACh and SNP) was attenuated when adjusting for established CV risk factors, and no associations were observed in conduit arteries by forearm FMD and post-ischemic reactive hyperemia and eGFR^48^. In agreement with this, our results in patients with hypertension and a wide range of eGFR suggest that increased levels of circulating eGCX markers is related to structural and functional vascular changes independent of eGFR.

To further elaborate the impact of kidney function on measured circulating vascular biomarkers, separate analyses to investigate relations between biomarkers and eGFR and uACR were performed. Our results show that kidney function was an independent determinant only for VCAM-1. If this is due to higher concentrations by loss of excretion, or by upregulated production of VCAM-1 cannot be determined from our current study.

There are some important strengths of our study. First, we simultaneously measured circulating vascular biomarkers and physiological assessment of vascular function in several vascular beds, which controls for potential fluctuations in confounding factors. Second, we studied patients with a wide range of age and eGFR, also including patients with moderate CKD, a population not well studied. This broadens the generalisability of our results. However, our study has notable limitations. First, the definition, selection, and application of biomarkers is critical^27^. We studied a limited number of circulating biomarkers of vascular endothelial function and markers of eGCX proteins. Additional techniques to investigate the eGCX layer would have been of added value. Of note, activation of soluble circulating biomarkers indirectly represents signs of structural and functional vascular changes as endothelial dysfunction and should therefore not be considered interchangeable with physiological measurements of vascular function, which might explain the weak associations. Second, the sample size limited the number of potential confounding factors to be evaluated by multivariable analyses.

In conclusion, soluble circulating eGCX markers HA and SDC-1 were independently related to physiological indices of aortic stiffness, endothelial function of smaller arteries, and skin microvascular endothelial function in hypertensive patients with impaired renal function. However, no investigated circulating vascular biomarker was independently related to physiological indices of large artery endothelial function or measures of left ventricular diastolic function. All circulating vascular biomarkers showed weak relations to physiological measurements and should therefore not be considered as proxies for physiological measures of vascular function and structure.

Future studies should explore whether combining assessment of vascular function by circulating biomarkers and physiological measures could improve early detection and risk stratification of vascular and renal disease progression.

## Methods

### Study design and subjects

This study combines cross-sectional data from two randomized clinical trials, the Doxazosin-Ramipril Study (DoRa), and the Sympathetic Activation and Inflammation in Moderate Kidney Failure and in Diabetic Nephropathy: Disease Modification with Vitamin-D Receptor Activation – the SOLID Trial (SOLID)^49,50^. Main results have been published elsewhere. In brief, DoRa investigated the effects of blocking the renin-angiotensin system on endothelial function, arterial stiffness, and on hemostasis^49,51^. In SOLID the primary aim was to evaluate the effects of treatment with active vitamin D on sympathetic activation and vascular function in non-diabetic CKD patients^50^. In DoRa, all study subjects were untreated, with no antihypertensive medication or statin treatment. Subjects in SOLID had ongoing antihypertensive treatment, where a majority were on ACE inhibitors or angiotensin receptor blockers, as presented in Table 1.

### Cardiovascular assessments

Study subjects were examined at the Cardiovascular research laboratory, Danderyd University Hospital, Stockholm (Sweden) following a standardized vascular protocol after overnight fasting with no intake of caffeine, nicotine substances, or intake of current medication, to avoid influence on endothelial function. For details, see elsewhere^49,50^.

Brachial BP was obtained as a mean of 3 readings 1 min apart on the right arm with an appropriately sized cuff by an oscillometric device (OMRON 705IT, OMRON Healthcare Co Ltd, Kyoto, Japan). MAP was calculated as diastolic + 1/3 (systolic – diastolic BP). Pulse pressure was calculated as systolic – diastolic BP. PWA was evaluated by the SphygmoCor device (AtCor Pty, West Ryde, NSW, Australia) with two-site applanation tonometry (Millar Instruments, Houston, TX, USA) to measure AIx, central systolic and diastolic BP, cPP, and cfPWV according to recommendations^52^, as described elsewhere^49,50^.

Large artery endothelial function was assessed by forearm FMD and post-ischemic reactive hyperemia by measuring the relative change in diameter of the brachial artery before and after cuff deflation, using the Vivid 7 Dimension ultrasound device (GE Medical System, Horten, Norway) according to recommendations^53^, as described previously^49^. Endothelium independent vasodilatation was induced by 0.4 mg GTN given sublingually (Nitrolingual, G Pohl-Boskamp GmbH & Co KG, Hohenlockstedt, Germany). Relative changes in artery diameter were calculated from rest to 4 min following GTN administration. The inter-assay coefficient of variation for FMD in our laboratory is 15% (*n* = 20).

Endothelium-dependent vasodilation of smaller resistance arteries (data only available for DoRa) was evaluated by applanation tonometry and PWA with beta 2-adrenoceptor agonist stimulation (terbutaline 0.25 mg sc; Bricanyl, AstraZeneca, Mölndal, Sweden), as described elsewhere^49^. In brief, the RIΔ%, the relative change of height of the reflecting diastolic radial pulse waveform before and after stimulation), was taken as a marker of endothelium-dependent vasodilation of the resistance arteries^14^.

To evaluate skin microvascular reactivity we studied endothelium dependent and independent vasodilation (data only available for DoRa) by laser Doppler fluxmetry (Periflux system 5000, PF 5010 LDPM Unit, PF5010 Temp Unit, and 481-1 Single Probe, Perimed, Järfälla, Sweden), using transdermal iontophoretic drug administration of ACh (Sigma-Aldrich AB, Stockholm, Sweden) and SNP(Hospira, Inc., Lake Forest, IL, USA) in small electrode chambers placed on the volar side of the forearm. The procedure has been described elsewhere^49^. First, basal blood flow was registered, expressed as perfusion units (PU). Second, local drug application was induced by using a small electrical current for 60 s, with continuous registration during 15 min to detect the maximum peak flux for ACh and SNP, respectively.

Transthoracic echocardiography and pulsed Doppler echocardiography (Vivid 7 Dimension, GE Medical System, Horten, Norway) was performed according to current recommendations^54,55^, as described elsewhere^38^. Left ventricular (LV) diastolic function was assessed by E/e’ and left atrial volume index (LAVI). Body mass index was calculated as weight/height^2^.

### Biochemistry

Fasting blood samples were collected from an antecubital vein after a 20 min period of supine rest, using Eclipse blood collection needles (21 G x 1–1/4”) and Vacutainer tubes (Becton Dickinson Co, Cedex, Meylan, France) containing sodium citrate (3.8%) or EDTA (1.8 mg/ml blood), as appropriate. The samples underwent immediate centrifugation at x 2000 g at 20 °C for 20 min and were then aliquoted and stored at -80 °C until further analysis.

Commercially available enzyme immunoassays (ELISA) were used to determine soluble eGCX markers [SDC-1 (Abcam plc, Cambridge, United Kingdom) and HA (Quantikine ELISA, R&D systems, Europe Ltd] and soluble adhesion molecules [E-selectin (Quantikine ELISA, Bio-Techne Ltd, UK), for ICAM-1 and VCAM-1 (MSD Multi-spot Assay System, Mesoscale diagnostics, LLC, USA]. EDTA plasma was used to analyse leukocyte and platelet counts using an automated blood cell counter (Technicon H1, Hematology System; Technicon Instruments Corp, Tarrytown, NY, USA). Routine biochemistry was analyzed by standard procedures and fasting blood samples. eGFR was calculated by the CKD-EPI formula. Low density lipoprotein cholesterol (LDL) values were calculated by the Friedewald formula as total cholesterol – plasma HDL – (0.45 *x* fasting plasma triglycerides). Albuminuria was analyzed by standard procedures as the uACR.

### Statistics

Data are presented as mean values ± SD or median and interquartiles. Bivariate linear regression and Pearson’s correlation coefficients (r) were used to investigate the relationship between circulating vascular biomarkers and physiological methods assessing vascular function. Blood vascular biomarkers were log transformed to achieve normal distribution. For bivariate correlations with probability (p) ≤ 0.05, multivariable linear regression analyses were performed including age, sex, MAP, ongoing antihypertensive medication and eGFR. To evaluate predictors of elevated aortic stiffness, two multivariable logistic regression models were constructed using recommended clinical cut-off values of cfPWV^32^, and median-based cfPWV stratification for comparison, using the same independent variables and covariates as the linear model.

The significance level was set to a (*p*) of < 0.05 for the final multivariable model. The statistical program used was SPSS version 28 (IBM Corp. IBM SPSS Statistics for Windows. Armonk, NY, USA).

## Supplementary Information

Below is the link to the electronic supplementary material.

Supplementary Material 1

## Data Availability

The datasets used and/or analyzed during the current study available from the corresponding author on reasonable request.
