# Supplementary material for: Circulating vascular biomarkers in relation to physiological indices of aortic stiffness and endothelial function in hypertension
Source: Sci Rep. 2025 Oct 28;15:37733. doi: 10.1038/s41598-025-25475-6 (PMC12568971; doi:10.1038/s41598-025-25475-6)
Supplement: Supplementary file 1 — Supplementary Material 1 [file 41598_2025_25475_MOESM1_ESM.docx]

| Variable | Estimate | SE | *p*-value | OR | 95% CI |
| --- | --- | --- | --- | --- | --- |
| Intercept | –33.50 | 9.23 | 0.0003 | – | – |
| Age (per year) | 0.310 | 0.081 | <0.0001 | 1.36 | 1.16–1.60 |
| MAP (per mmHg) | 0.116 | 0.047 | 0.0142 | 1.12 | 1.02–1.23 |
| Sex (female vs male) | –1.10 | 0.51 | 0.0311 | 9.10 | 1.22–67.72 |
| HA | –0.030 | 0.052 | 0.5565 | 0.97 | 0.88–1.07 |
| eGFR | 0.00008 | 0.030 | 0.9980 | 1.00 | 0.94–1.06 |
| Treatment | 0.404 | 0.896 | 0.6521 | 0.45 | 0.01–14.97 |

**Supplementary Table 1. Parameter estimates and odds ratios from logistic regression model predicting cfPWV ≥10 m/s**

cfPWV, carotid to femoral pulse wave velocity; MAP, mean arterial pressure; HA, hyaluronan; eGFR, estimated glomerular filtration rate

**Supplementary Table 2. Parameter estimates and odds ratios from logistic regression model predicting cfPWV ≥8.6 m/s**

| Variable | Estimate | SE | *p*-value | OR | 95% CI |
| --- | --- | --- | --- | --- | --- |
| Intercept | –13.19 | 4.51 | 0.0034 | – | – |
| Age (per year) | 0.163 | 0.044 | <0.0001 | 1.18 | 1.08–1.28 |
| MAP (per mmHg) | 0.037 | 0.030 | 0.2184 | 1.04 | 0.98–1.10 |
| Sex (female vs male) | –0.30 | 0.31 | 0.3198 | 1.83 | 0.56–6.06 |
| HA | –0.025 | 0.042 | 0.5407 | 0.97 | 0.90–1.06 |
| eGFR | –0.0025 | 0.026 | 0.9206 | 1.00 | 0.95–1.05 |
| Treatment | 0.404 | 0.794 | 0.6110 | 0.45 | 0.02–10.01 |

cfPWV, carotid to femoral pulse wave velocity; MAP, mean arterial pressure; HA, hyaluronan; eGFR, estimated glomerular filtration rate
